# Multicellular Liver Organoids: Generation and Importance of Diverse Specialized Cellular Components

**DOI:** 10.3390/cells12101429

**Published:** 2023-05-19

**Authors:** Giuseppe Ietto, Valentina Iori, Mattia Gritti, Davide Inversini, Angelita Costantino, Sofia Izunza Barba, Z. Gordon Jiang, Giulio Carcano, Daniela Dalla Gasperina, Giuseppe Pettinato

**Affiliations:** 1 General, Emergency and Transplant Surgery Department, ASST-Sette Laghi, 21100 Varese, Italy; 2 Department of Medicine and Innovation Technology (DiMIT), University of Insubria, 21100 Varese, Italy; 3 Department of General Surgery, Humanitas Clinical and Research Center, Rozzano, 20089 Milan, Italy; 4 Department of Drug and Health Sciences, University of Catania, 95124 Catania, Italy; angelita.costantino@unict.it; 5 Division of Gastroenterology, Department of Medicine, Beth Israel Deaconess Medical Center, Harvard Medical School, Boston, MA 02215, USA; 6 Department of Infectious Diseases, ASST-Sette Laghi, 21100 Varese, Italy

**Keywords:** endothelial cells, hepatic differentiation of hiPSCs, liver bioengineering, liver organoids, multicellular organoids

## Abstract

Over 40,000 patients in the United States are estimated to suffer from end-stage liver disease and acute hepatic failure, for which liver transplantation is the only available therapy. Human primary hepatocytes (HPH) have not been employed as a therapeutic tool due to the difficulty in growing and expanding them in vitro, their sensitivity to cold temperatures, and tendency to dedifferentiate following two-dimensional culture. The differentiation of human-induced pluripotent stem cells (hiPSCs) into liver organoids (LO) has emerged as a potential alternative to orthotropic liver transplantation (OLT). However, several factors limit the efficiency of liver differentiation from hiPSCs, including a low proportion of differentiated cells capable of reaching a mature phenotype, the poor reproducibility of existing differentiation protocols, and insufficient long-term viability in vitro and in vivo. This review will analyze various methodologies being developed to improve hepatic differentiation from hiPSCs into liver organoids, paying particular attention to the use of endothelial cells as supportive cells for their further maturation. Here, we demonstrate why differentiated liver organoids can be used as a research tool for drug testing and disease modeling, or employed as a bridge for liver transplantation following liver failure.

## 1. Introduction

The number of patients dying from end-stage liver disease and liver failure in the United States is over 40,000, with another 2000 suffering from acute fulminant hepatic failure [1]. The only viable therapy currently available to these patients is liver transplantation, in most cases. In the Western world, 96% of transplanted livers come from deceased donors. Unfortunately, the current donor liver supply cannot meet the demand for transplants and many patients die on the waiting list.

For a long time, people have been looking for alternatives to liver transplants. Several studies have been conducted using hepatocytes for the evaluation of drugs and to model liver diseases, as well as for potential use in transplantation. However, human primary hepatocytes (HPH) cannot address the massive shortage of donor liver organ supply as they neither expand nor grow for an extended period of time in vitro [2]. Moreover, HPH are extremely sensitive to cold storage injury, making cryopreservation difficult if not impossible [3]. 

Human-induced pluripotent stem cells (hiPSCs) are a promising option in regenerative medicine due to their pluripotency, high proliferation capacity, and lack of ethical controversy. Adding stemness factors allows patient-derived cells to be retro-engineered into pluripotent cells [4,5,6]. Differentiating hiPSCs into liver organoids has been proposed as a cell therapy strategy for liver failure, bioengineered livers, drug testing, and liver disease modeling [7]. Several research studies have demonstrated the potential of hiPSC-derived liver organoids [8,9]. These organoids are capable of secreting human albumin and alpha-1-antitrypsin (A1AT), synthesizing urea, and regulating cytochrome P450 (CYP) enzymes in vitro, among other liver functions [8,9]. However, in most cases, these organoids lack any endothelial component, which represents an important part of liver organogenesis in vivo [10,11]. Nonetheless, the broader application of hiPSC-derived liver organoids demands better scalability, an improved post-differentiation phenotype, and proven long-term function both in vitro and in vivo [12,13,14]. 

Herein, we present a critical review of recent research on liver organoids derived from hiPSCs and technologies for human embryoid bodies generation. Human embryoid bodies are three-dimensional (3D) cell aggregates that emulate in vitro the embryonic development that occurs during in vivo organogenesis. This 3D structure is essential as it allows adequate cell polarization and tissue organization of the differentiating organoid, as well as the possibility to include additional supportive cells, such as endothelial cells, among other cell types. 

## 2. Generation of Embryoid Body for Three-Dimensional Culture

HPH dedifferentiation following adherence cell culture (2D culture) is a well-known process that is followed by the loss in hepatocyte function, such as reduced plasma protein synthesis (i.e., albumin) and loss of detoxifying abilities [15]. Adherence culture constrains adhering cells to adopt a flattened cytoskeleton shape, which restricts cell-cell and cell-matrix interactions, reducing cell polarization and disrupting the signaling pathways necessary for proper hepatocyte activity [15]. Such modifications are particularly relevant to hepatocytes, which are polygonal with multi-polarized structures including at least two basolateral and one apical surface [16]. Preserving liver function ex vivo requires fully functioning hepatocytes to be used for primary hepatocyte transplantation, also for toxicological screening, and in the development of bioartificial liver devices [15]. 

The high-throughput differentiation of hiPSCs into a specific cell lineage is critical for clinical application, especially when large numbers of cell populations are required. A commonly used strategy for initiating the differentiation of hiPSCs into organoids involves the generation of human embryoid bodies (hEBs). hEBs are composed of a three-dimensional cell aggregate that resembles the native embryonic structure [17]. They allow the differentiation of tissues derived from all three germ layers, which can be guided by different factors, such as the medium composition, the number of cells composing the hEBs, i.e., their sizes [18,19,20,21]. For example, small EBs cannot sustain differentiation, whereas larger EBs may result in central core necrosis [21]. Based on current developments, EBs can be generated using the following methods: (i) spontaneous self-aggregation in non-adhesive wells/dishes under static conditions [22], (ii) hanging drop culture, where small numbers of stem cell colonies are included in a 30 μL drop to form a cluster [23], (iii) agitation culture (rotary culture, rocking culture, bioreactors) [24] or (iv) microcavities and agarose micromolds [25,26,27,28]. 

Conventional procedures for EB generation involving the mechanical dissociation of stem cell colonies have resulted in EBs with variable sizes, leading to heterogeneous organoid differentiation [29]. To ensure synchronized EBs formation, it is preferable to employ singularized hiPSCs, which permit precise control of the cell seeding density in each EB to regulate their dimensions and consistency. Multiple bioreactors have been created to produce hEBs to achieve accurate, scalable differentiation [30,31]. The transplantation of differentiated cells utilizing bioreactors has not demonstrated any advantages for tissue replacement, despite the benefits of this approach with the scalable production of organoids [32]. However, this technique suffers from several disadvantages, such as the heterogeneity between the obtained hEBs, which does not allow for the uniform differentiation process, and the overall extended time needed for the overall generation of organoids [32].

When hiPSCs colonies are dissociated into single cells, the disruption from direct cellular contact exposes them to the susceptibility of apoptosis, resulting in a lower rate of hEB production [33]. Rho-associated kinase (ROCK) inhibitor Y-27632 has been used to maintain the survival of singularized hiPSCs, presumably by blocking anoikis or increasing cell-cell interaction to enable their aggregation [33]. Although the ROCK inhibitor (ROCK-i) promotes the re-aggregation of singularized hiPSCs, the use of liver organoids as a therapeutic approach might be precluded by this small molecule [34]. Centrifugation is another way of aggregating a single hiPSCs suspension (i.e., the spin EB method) [35]. However, this procedure may damage the hiPSC and limit the automated, scalable development of hEBs [36]. In order to manufacture homogenous hEBs from dissociated hiPSCs without employing ROCK-i or centrifugation, our team developed a unique technique involving the use of an agarose micromold [26,27]. We achieved homogenous and synchronized hEBs in a scalable fashion by precisely controlling the cell seeding density. Beginning with a homogenous pool of EBs to allow for a more synchronous differentiation, so that all hEBs similarly responded to diverse growth inputs.

## 3. Liver Regeneration and the Importance of Replicating Its Structure When Differentiating Organoids

The regenerative abilities of the liver make it unique among all of the human organs. After being injured, either by diseases or resection, the liver can regenerate and maintain its original tissue mass. Higgins and Anderson (1931) were the first to investigate liver regeneration in an animal model, where a two-thirds partial hepatectomy (PHx) was performed without harming the other lobes [37]. Since this landmark study, the source of cells responsible for tissue regeneration, growth, and maintenance have long been prime topics of research. In this regard, there are two major hypotheses that try to explain the modality from which the liver regenerates: one proposes that most hepatocytes have an equivalent ability to regenerate despite their position [38]; and the alternative hypothesis argues that the position of hepatocytes within the liver dictates their regenerative capacity [39]. 

**Figure 1:**
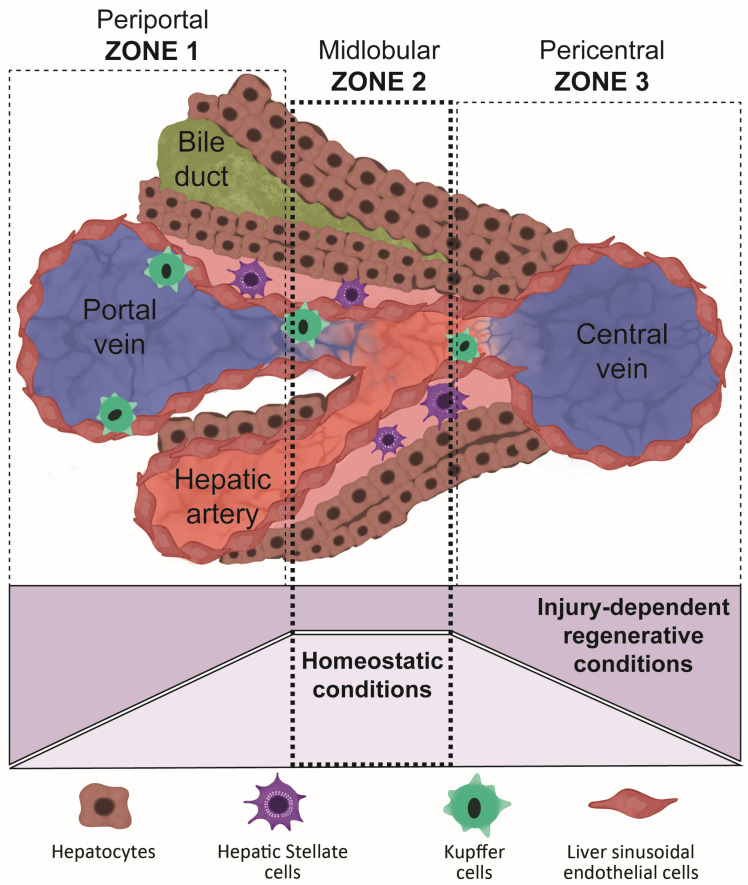
Organization of the main liver structures called lobules. This scheme shows the lobule subdivision into three zones from the portal vein (periportal zone 1), to a midlobular area (midlobular zone 2), and reaching the central vein (pericentral zone 3). Each zone differs in terms of its sensitivity to homeostatic perturbations.

The human liver is organized into fundamental structural units called the liver lobules, in which hepatocytes express and produce distinctive metabolic and synthetic proteins. The liver lobules are divided into three zones between portal and central veins, driven by the nutrients and oxygen gradient (Figure 1). Recent studies have highlighted that hepatocytes residing in the mid-lobular (zone 2) are responsible for liver regeneration in homeostatic conditions [39]. This is potentially because mid-lobular hepatocytes are protected from toxic injuries that occur in zone 1 and ischemic injuries in zone 3 [39]. 

In the case of liver resection, studies have demonstrated that the restoration of the liver mass is mainly due to the compensatory hypertrophy and hyperplasia of the remaining hepatocytes [40,41]. 

Changes in portal pressure following hepatectomy could be a mechanism that triggers liver regeneration [11,42]. However, liver tissue regeneration, following hepatectomy, lead to a more faithful restoration of the microenvironment structure rather than the macro-architecture. Such a regenerated microstructure shows complete functional activity that resembles the one in the native liver. Failure to replicate the macroscopic anatomy of the liver during its regeneration may be responsible for the production of fibrotic tissue over time [43,44].

Following these studies in liver regeneration, together with the embryological cues during liver organogenesis, scientists can select growth factors shown in liver generation and liver embryogenesis, to facilitate the development of ex vivo liver organoids. An example of such factors is the hepatocyte growth factor (HGF). Its production is augmented during the early phase of liver regeneration by non-proliferative LSEC. An increase in the secretion of Id1 through the VEGFR2/VEGFA pathway leads to an active secretion of HGF and Wnt, but a reduction of the hepatocyte growth inhibitor TGF-β and angiopoietine-2 [45].

Other crucial humoral factors in response to the regenerative stimuli in the liver cells are the urokinase plasminogen activator (uPA) and its downstream effector plasminogen, which, through the cleavage of the pro-HGF and the extracellular adenosine triphosphate (ATP), allows the uPA to trigger the c-Jun–amino-terminal kinase (JNK) pathway, with the consequent induction of early genes FOS and JUN, that in turn initiate the AP-1 DNA-binding activity [44,46]. This cascade leads to hepatocyte replication activity, starting the regenerative process.

Another important differentiation cue is the Wnt/β-catenin pathway that has been shown to play a pivotal role during in vivo liver differentiation. This pathway is first activated at the beginning of the liver bud formation and is then suppressed during the differentiation from hepatoblasts to hepatocyte [8]. The inhibition of this pathway using specific Wnt inhibitors, such as WIF-1 and DKK-1, are essential to lead the generation of mature hepatocyte-like cells [8].

The use of such growth and mitogenic factors that we learned throughout the scientific analysis of liver regeneration are not the only part that needs to be taken into consideration when we talk about liver organoids generation. As mentioned above, the microstructure of the liver should also be recapitulated within a liver organoid. Given the fact that the liver is composed of highly specialized cellular components, with a specific spatial distribution, to replicate it in vitro is complex, so it is important to use multicellular components in the same organoid. Such cellular components, once in the presence of each other, could potentially self-organize to generate liver lobule-like structures, supporting a more detailed physiological representation of microtissue. In our experiments, mixing differentiating hiPSC-derived hepatocytes with primary LSEC and hepatic stellate cells, we were able to demonstrate a topological disposition of such cells as shown in the real liver. This promising result could lead to the generation of a more faithful in vitro liver model. Moreover, the addition of the native liver extracellular matrix into the differentiating organoid could potentially instruct the specialized cells within the multicellular organoid to position themselves in a similar disposition as seen in the liver lobules.

Another critical component to consider in liver organoid generation is how they would be able to properly restore liver function. Liver organoids are small masses, as compared to the massive size of the human liver; however, their capability to substitute liver function does not reside in their mass, but rather in their ability to repopulate the diseased organ once transplanted into a patient. In our previous studies, we demonstrated how our liver organoids were able to repopulate the host liver after intrasplenic transplantation [8]. Others have shown primary hepatocytes could be used in a similar manner in a clinical setting [47]. The scalable production of high-functioning liver organoids could provide an alternative to orthotropic liver transplant, similar to the transplantation of pancreatic islets.

## 4. Differentiation Strategies

Liver organoids from hiPSCs must exhibit similar morphology and function to primary hepatocytes before they can be considered for therapeutic applications. In the last decade, many differentiation procedures for creating liver organoids from hESCs and hiPSCs have been reported [8,13,48,49,50,51,52]. Each of these research groups concluded that homogeneous differentiation relies on precise control of the culture conditions, differentiation protocols, and scalability. Table 1 displays the principal differentiation techniques used for generating liver organoids from hESCs and hiPSCs.

The most effective method is to regenerate the in vivo signaling pathways during embryogenesis in an in vitro setting. The fetal liver develops in three stages. First is the generation of the definitive endoderm (DE), followed by the production and proliferation of hepatoblasts, and finally, the differentiation of hepatoblasts into mature, functioning hepatocytes. Hepatoblasts are bi-potent progenitor cells that can differentiate into both hepatocytes and cholangiocytes [59]. This process, which finally leads to liver organogenesis, is driven by a cascade of signaling events in vivo. Specifically, the nodal, bone morphogenetic protein (BMP), and activin signaling pathways promote the specification of the mesendoderm, from which the mesenchyme and endoderm develop [53,60]. In addition to activin-A, the up-regulation of additional pathways, such as the fibroblast growth factor (FGF) and Wnt signaling, have been shown to stimulate endoderm development. Some published methods require low serum concentrations for activin-A to promote endoderm development [13,53,60,61,62,63]. Further signals from the FGF and BMP families, particularly BMP4, FGF2, and FGF4, induce hepatoblast differentiation [13,64,65]. After the development of the liver bud, the hepatocyte growth factor (HGF) and oncostatin induce the differentiation of hepatoblasts into mature hepatocytes [66].

In spite of the use of specific growth factors naturally observed in hepatogenesis to differentiate hiPSCs, no liver differentiation protocols have addressed the Wnt/β-catenin pathway downregulation as of yet, which is an important step during in vivo liver development [56,67,68,69]. The Wnt/β-catenin pathway is ubiquitous across species in cell differentiation into particular lineages, including hepatocytes [70], and its influence on liver embryogenesis is highly time-regulated [71,72]. During the initial phases of liver development, β-catenin expression increases between E10 and E12, and then declines after E16. The Wnt pathway’s regulation emerges later in cell differentiation and, in conjunction with β-catenin, is essential for differentiating liver progenitor cells (i.e., hepatoblasts) into hepatocytes or cholangiocytes. Once active, it directs hepatoblasts onto cholangiocytes; if it is inhibited, it directs hepatoblasts toward hepatocytes [73,74]. Using these characteristics, it is conceivable to modify the fate-determining hepatobiliary stage during differentiation to boost the yield of either cell phenotype. By including Wnt/β-catenin pathway inhibitors, it is possible to adjust the ratio of hepatocytes to cholangiocytes, enhancing the hepatocyte output [73,74,75,76].

Our team has devised a technique that incorporates multiple Wnt/β-catenin signaling inhibitors, Wnt inhibitory factor-1 (WIF-1) and dickkopf-1 (DKK-1), which has allowed us to increase the liver differentiation much further than that obtained by existing methodologies [8,13]. Our differentiated liver organoids display many characteristics of human primary hepatocytes, including the polygonal shape and multinucleated cells. After 48 h in culture, they release multiple essential hepatic proteins determined by the enzyme-linked immunosorbent test (ELISA). Human albumin concentrations in 5 × 10^5^ cells ranged from 120 to 130 ng/mL, corresponding to approximately 60% of albumin synthesis by human primary hepatocytes (128 ng/mL vs. 199 ng/mL, *p* = 0.0009; AFP: 0.18 ng/mL vs. 0.19 ng/mL, *p* = 0.69; fibrinogen: 0.062 vs. 0.064, *p* = 0.0015). The functional properties were equivalent to those performed by human primary hepatocytes, including acetylated low-density lipoprotein (DiI-ac-LDL) uptake, indocyanine green (ICG; Cardiogreen) absorption, and release after 6 h, glycogen storage by periodic acid-Schiff (PAS) staining, and the cytoplasmic accumulation of neutral triglycerides and lipids by oil red staining. Using the P450-GloTM assay, we determined that our differentiated liver organoids reacted to inducers based on the rise in the activity of three isoforms of cytochrome P450 (CYP1A2, CYP3A4, and CYP2B6). This detoxifying profile is found at a lower degree of induction than in primary human hepatocytes (CYP3A4: 67 vs. 82, *p* = 0.0232; CYP2B6: 14 vs. 98, *p* < 0.0001; CYP1A2: 22 vs. 98) [8,13]. When we transplanted our differentiated liver organoids into a rat model of acute liver failure, their survival rate dramatically improved, and human albumin was found in the rat serum [13].

In addition to soluble factors, hESCs and hiPSCs can be differentiated into liver organoids by the directed expression of transcriptional factors necessary for liver organ development. Since the 2007 production of hiPSCs by Yamanaka’s team, other groups have been able to directly convert somatic and embryonic stem cells into hepatocyte-like cells (HLCs), skipping the pluripotent stem cell step. Huang et al. pioneered the generation of HLCs from mouse fibroblasts (MEFs), showing that the transduction of these cells from p19arf ^−/−^ mice using GATA4, HNF1β, and FoxA3 promoted the formation of hepatic-like cells that also showed the presence of hepatic markers and recovered liver function in a mouse model after transplantation [67]. Simultaneously, Sekiya et al. employed a combination of transcriptional factors (HNF4α, FoxA1, FoxA2, or FoxA3) to convert MEFs into HLCs, demonstrating that the resulting cells increased animal survival by 40% 10 weeks after cell transplantation [77]. Furthermore, two more groups have documented the effectiveness of transduction utilizing alternative transcriptional factors [68,78,79]. Notably, Nakamori et al. created more mature human HLCs by overexpressing activating transcription factor 5 (ATF5), CCAAT/enhancer-binding protein alpha (c/EBPa), and Prospero homeobox protein 1 (PROX1) [68]. The transduction of these molecules upregulated numerous liver cell markers, such as drug metabolism enzymes and hepatic transporters. Yahoo et al. improved the hepatic lineage of mESCs by driving the expression of HNF1β and FoxA3, and by employing particular hepatic culture conditions [78]. This group also determined that the exogenous production of HNF4α during directed differentiation may be a suitable strategy for investigating the impact of overexpression on the hepatic differentiation of mESCs [79]. 

HLCs have been differentiated largely using human pluripotent stem cells, such as hESCs and hiPSCs, although other cell types have also been employed. Mesenchymal stem cells from several sources, including bone marrow, adipose tissue, skin, placenta, and umbilical cord, have been developed into HLCs with characteristics comparable to mature primary hepatocytes [80,81,82,83,84,85,86,87]. Using a four-step differentiation protocol, our team converted human bone marrow stem cells into HLCs, creating MSC-derived HLCs that could also restore liver function and enhance survival, compared to control rats [76]. Our MSC-derived HLCs in vivo after transplantation demonstrated the ability to synthesize human albumin, as highlighted by its presence in the recipient rat serum [88].

## 5. Two-Dimensional vs. Three-Dimensional Culture

The capacity to generate phenotypically normal cells from any tissue is contingent on the growth factor combination and the culture method employed during differentiation. Although monolayer cultures are appropriate for learning fundamental cell biology, cells cultivated with these approaches develop a flattened shape and experience altered cell-cell and cell-environment interactions. This structure alters stem cell pluripotency and differentiation by affecting the polarization and crucial signal transduction pathways [89]. Conventional hepatic differentiation techniques that rely on two-dimensional adherent culture have produced cell populations that lack all of the characteristics of primary hepatocytes [14,54,55,63]. 

Multiple cell types interact to form a three-dimensional structure in the liver bud during organogenesis [90,91,92]. The formation of cell-cell junctions, notably via E-cadherin, promotes hepatocyte development [93,94]. Primary hepatocytes and hiPSC-derived HLCs cultivated in three-dimensional media retain their hepatic characteristics more effectively than their counterparts generated in two-dimensional culture [95,96,97,98,99,100,101]. Some investigations employing hiPSCs have combined two- and three-dimensional cultures for the ultimate maturity of differentiated HLCs [8,13,56,57,99,102,103,104,105]. Three-dimensional culture-based differentiation using hiPSC-EBs has several advantages over monolayer culture-based differentiation, along with a greater ability for high cell density, by eliminating the cell-cell contact inhibition and growth characteristic of two-dimensional cultures and promoting the maturation of HLCs through cell-cell contact [106]. In monolayer cultures, differentiating cells may have quick and easy access to the growth factors in the medium.

**Figure 2:**
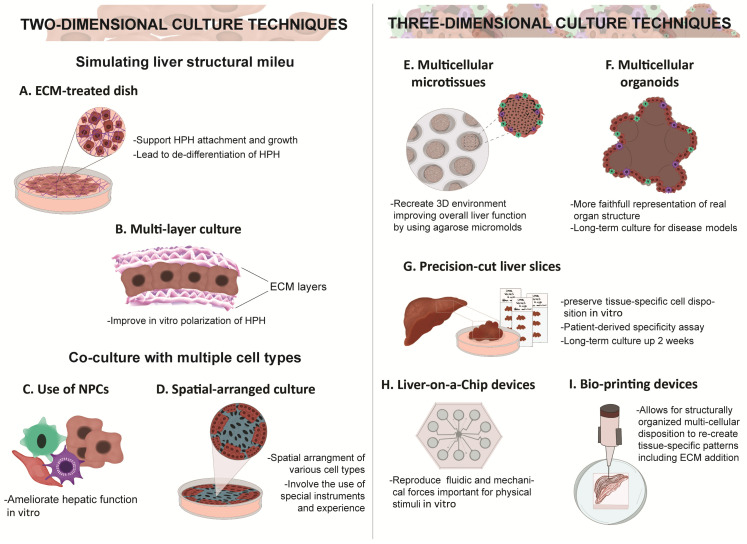
Schematic representation of different technologies that are used to reproduce the liver structure in vitro, both in a two-dimensional and three-dimensional setting. (**A**) Extracellular matrices (ECM) are used to improve hepatocyte growth and prevent de-differentiation processes. (**B**) Multi-layer culture using ECM allows for a better simulation of extracellular matrix embedding. (**C**) The use of non-parenchymal cells (NPCs) allows for the amelioration of hepatic function in vitro. (**D**) The use of micro-patterned surfaces leads to a better spatial arrangement of the cells, allowing for the recapitulation of liver architecture in two-dimensional culture. (**E**) Multicellular microtissue allows for recreating the three-dimensional organization that can be found in the real organ, improving the overall function. (**F**) Multicellular organoids are typically created from primary cell lines or tissue explants (e.g., biopsies) and allow for a better recapitulation of the organ function in vitro. (**G**) Precision-cut liver slides allow for the preservation of the original cell disposition in the liver, as well as the diversity of each cell component. (**H**) Liver-on-a-Chip devices can study mechanical and fluidic forces within the system that are missing in the other three-dimensional techniques; however, does not always allow for full recapitulation of the spatial organization of the cells. (**I**) Bio-printing devices allow for artificially reproducing biophysical structures to mimic the organ microenvironment, as well as the potential addition of ECM in the bio-ink.

Nonetheless, embryonic tissues originate via inductive signaling, determined by a growth factor gradient within a three-dimensional structure. Similarly, the distinctive three-dimensional structure of EBs may resemble the environment of the in vivo embryo, which may provide a favorable situation for reproducing gradient diffusion and the appropriate signaling for tissue differentiation in vivo. A disadvantage of EBs is the risk of core necrosis formation due to the inadequate diffusion of nutrients and oxygen at the cluster’s center [21]. The risk of core necrosis could be mitigated by refining the method to generate the EBs, and potentially be prevented by utilizing bioengineering technologies [26,28,107,108,109], supporting cells (e.g., endothelial cells) that facilitate nutrition exchange and engraftment following transplantation [88,110]. Table 2 outlines the cell types utilized as hepatocytes and to generate liver organoids for possible cell treatment. Figure 2 shows the multiple techniques used to improve liver function in vitro, both using two-dimensional and three-dimensional culture methods, plus various histological and bioengineering strategies.

## 6. Co-Culture Methods and Use of Extracellular Matrices

Several studies have investigated the use of co-cultures as supporting cells to enhance hepatic specification in differentiating hiPSCs, in order to reproduce the signal transduction pathways found during in vivo liver organogenesis. Pal et al. employed a conditioned medium from a human hepatocellular carcinoma (HepG2) cell line to differentiate hESCs into HLCs to explore the in vitro hepatic consequences of ethanol toxicity [111]. Across several studies, fibroblast cells generated from disparate sources (STO feeder cells, 3T3 cells, and pluripotent stem cell (PSC)-derived fibroblast-like cells) were employed as adjuvant cells to promote the liver differentiation of hESCs and hiPSCs in vitro [112]. Endothelial cells, mesenchymal cells, Kupffer cells, and stellate cells, which are involved in liver embryogenesis, have been integrated into differentiation methods to promote hepatic specification and maturation in HLCs [113,114,115,116]. 

The use of extracellular matrices or scaffolds that replicate the architecture of the developing liver in vivo is a second strategy for enhancing the in vitro hepatic differentiation of hiPSCs. Numerous studies have emphasized various artificial and natural matrices for promoting HLC differentiation, including collagen type I, vitrogen, matrigel, polyurethane foam [117], fibronectin [118], laminin, polyacrylamide [119], hollow fibers [120], poly-l-lactic acid plus polyglycolic acid [121], Ultra-Web nanofibers [122], alginate microbeads [123], nanofibrillar cellulose, and hyaluronan-gelatin [124]. Kanninen et al. used the HepaRG cell-derived acellular matrix to differentiate hiPSC-derived hepatic progenitor cells, showing the importance of how extracellular matrices can guide the differentiation processes [125,126,127,128]. 

Decellularization is an innovative method that combines scaffolds and extracellular matrices to repopulate a whole liver with native hepatocytes, hepatocyte cell lines (hepG2), or differentiated hiPSC-derived HLCs for drug screening and therapeutic applications. Decellularized livers or extracellular matrices from such livers have been adopted as three-dimensional regenerative scaffolds for the growth of primary hepatocytes. They also support long-term liver function and possess host-specific native liver structures [129]. After fatal hepatectomy, Skardal and coworkers showed that by transplanting primary hepatocytes grown with a synthetic hydrogel from tissue-specific extracellular matrices into rats they were able to restore liver function and dramatically improve the survival rate [130]. Geerts et al. recently devised a non-destructive approach for detecting cell loss during rat liver decellularization [131]. This group employed a strategy for decellularizing rat livers. Standard destructive methods were quality-controlled based on the DNA, collagen, and glycosaminoglycan (GAG) content of the scaffolds, as determined by histology. Computed tomography and perfusate analysis were also utilized as nondestructive decellularization monitoring alternatives. Consequently, they devised a method that yields scaffolds with much more GAG without compromising their cell removal efficacy. Mazza et al. developed such technologies, using the decellularized human liver as scaffolding for bioengineered livers [132]. They obtain recellularized cubical portions of an entire decellularized human liver with human cell lines, including hepatic stellate cells (LX2) and hepatocytes (Sk-Hep-1, HepG2). This study confirmed the biocompatibility of liver scaffold cubes subcutaneously implanted into immune-competent mice to avoid rejection. Although these new technologies are promising, they remain experimental using primarily immortalized or animal-derived cells. The use of synthetic extracellular matrices containing the same natural composition of integrin ligands found in human livers could be potentially used to create scaffolds that will naturally support the growth and differentiation of hiPSCs or mature primary cells, while maintaining an animal/human-derived free system.

## 7. The Role of Endothelial Cells in Organoid Differentiation and Vascularization 

In vivo organogenesis is a complex process that involves several factors. From the fertilization, passing through the gastrulation, many events occur, such as the embryonic germ layers formation, from which each organ or tissue will arise [133]. According to their disposition within the gastrula, three main germ layers can be distinguished: ectoderm, mesoderm, and endoderm. Each tissue or organ develops from one of these three germ layers, and often, the organogenesis occurs through the interaction of two or more adjacent layers [133].

It is difficult to fully reproduce this inductive process via an in vitro differentiation protocol [90,134]. To be suitable, an organoid should possess specific properties, such as possessing the same functional properties that relate to the original organ that it is intended to subsidize (e.g., albumin production for the liver organoids, insulin secretion for the pancreatic, etc.); avoiding immune rejection and properly engrafting into the host system after transplantation [135]. One must consider all of the different cues that each of these layers is putting together, and determine the doses and timing of each factor involved in the differentiation process. 

One important step common to all tissues and organs in our body during their in vivo development is the presence of endothelial cells that represent one of the first organized structures within the arising embryo, together with the heart and neural system [136,137]. Endothelial cells not only serve as the building blocks for blood vessels, through which the developing organs can obtain oxygen and nutrients, but they also actively participate in the formation of the arising organ by secreting differentiation factors called “angiocrine factors”, which are tissue-specific, and help the organ to properly develop [138,139,140,141]. Including endothelial cells in developing organoids is a strategy that has been used for over a decade [142]. By adding these endothelial cells, we can potentially recreate a biological niche that resembles the in vivo microenvironment [138]. It potentially brings crucial differentiation signals to the developing organoid that is otherwise missing from the culture media. One question that needs to be posed is, “which kind of endothelial cells” should be used during a particular type of organoid generation. Considering that every organ in the body possesses a specialized endothelial cell (sinusoidal cells in the liver, glomerular capillaries in the kidneys, etc.), we must ask if we should use the tissue-specific endothelial cell for each of the different organoids. 

For simplicity, several studies have used human umbilical vein endothelial cells (HUVEC) during the in vitro differentiation processes, as they are easy to be isolated from the umbilical cord, and they do not create any ethical controversy, and because they can be isolated and stored from their own umbilical cord at birth [142]. Such cells have demonstrated a degree of ability to improve the differentiation process and facilitate subsequent engraftment after transplantation [142]. However, the lack of tissue specificity of these cells might not fulfill the purpose of liver differentiation [138,139,140,141]. Another important question that needs to be asked is if these tissue-specific endothelial cells, once in the adult tissue, can still be able to secrete angiocrine factors useful for the differentiating organoid to properly develop. It might be that, once the organ has reached its maturation, the endothelial cells within it could lose their potential to drive the differentiation process; therefore, not the most optimal to promote parenchymal cell differentiation. In the case of the liver, studies have shown that following homeostatic regulations, angiocrine signals regulate the expansion of hepatocytes by allowing the propagation of diploid Axin2- and T-box transcription factor 3 (Tbx3)-positive cells, repopulating the liver [143]. These diploid cells are situated in proximity to the endothelial cells of the central vein in the liver. The production of angiocrine factors, such as Wnt2 and Wnt9b, from these specific endothelial cells preserves Axin2 and Tbx3 double-positive hepatocytes that eventually generate distal non-pericentral hepatocytes [139]. Moreover, the expression of specific angiocrine factors of Rspondin3 by endothelial cells situated in the central vein of the liver creates a β-catenin-dependent metabolic zonation while improving the regenerative capacity of the liver hepatocytes [144].

Furthermore, how mature endothelial cells could be isolated from patients is not clear; for example, if the use of pancreatic endothelial cells surrounding the islets of Langerhans is needed, they should be isolated, avoiding any risk of pancreatitis in the patient. Moreover, if there is a need to replace a specific organ, most likely, the structure of this organ is already compromised, including the endothelial cells in it, making them inadequate for our purpose. Thus, all of the above factors could impair the use of such tissue-specific endothelial cells, forcing the researcher to choose a type of endothelial cell that could be considered universal.

We have demonstrated that human adipose microvascular endothelial cells (HAMEC) can possess the appropriate features to be used as universal endothelial cells. These cells can be recovered from the patient’s adipose tissue with minimal risk. Using our three-dimensional culture system [26,27,28], we have shown that their addition into our differentiating liver organoids improved hepatocyte maturation by increasing the production and secretion of liver-specific proteins, such as albumin, among others [58,145,146]. Our liver organoids with HAMEC showed a strong response of liver phase I and II detoxification enzymatic activity, such as CYP1A2, 3A4, and 2B6, as well as resorufin conjugation, together with the ability to synthesize urea as a consequence of ammonium metabolism [58,145,146]. Storage abilities for glycogen, lipids, Ac-LDL, and ICG were also shown in our liver organoids. The presence of HAMEC displayed the ability to secrete specific coagulation factors normally produced by the endothelial cells, allowing for a comprehensive coagulation function of our liver organoids in vitro [58,145,146]. 

Increasing the post-transplantation vascularization to allow for the accelerated engraftment of the organoid is another important function that endothelial cells should possess [147]. This process can be promoted by the secretion of angiogenic factors from the endothelial cells within the organoid into the host tissue [147]. The recruitment of blood vessels from the surrounding tissue where the organoids are transplanted would be essential for successful transplantation. We showed that the inclusion of HAMEC improved the post-engraftment after transplantation, with 80% of the transplanted animals that received liver organoids composed of differentiated hiPSCs mixed with HAMEC, showing the presence of human albumin for more than 14 days in their serum, indicating that the uniform integration of HAMEC and hiPSCs can maintain the functions of transplanted cells [58,145,146]. 

**Figure 3:**
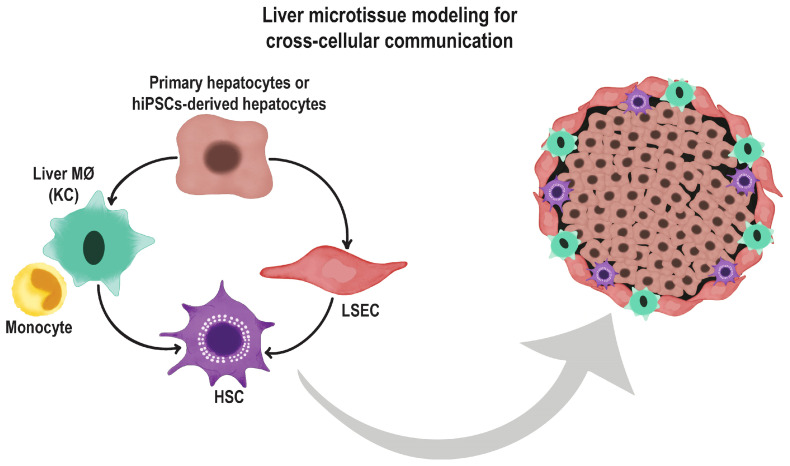
Schematic representation of how a liver microtissue can reproduce the cell composition of the real tissue. Liver sinusoidal endothelial cells (LSEC); Human stellate cells (HSC); Kupffer Cells (KC); Liver macrophage zero (MØ).

Organoids represent an incredibly immense source for cell replacement therapy, drug screening, and disease modeling, both in vitro and in vivo. However, these models do not always fully reproduce the original organ’s functions. This is especially true when following encouraging outcomes in pre-clinical models using differentiated organoids; the results in clinical trials do not always reflect the same success. An explanation of these discordant results can be found in the fact that disease onset and progression implicate the interaction between various cell lineages within the same organ. Therefore, the presence in the same organoid of all these cell types that participate in the development of a specific disease becomes of utmost importance to fully recapitulate any aspect of the diseased organ [148,149,150,151,152,153,154,155]. Organoids provide a better representation than primary cells in culture, as they recapitulate a phenotype closer to the in vivo condition, allowing for a higher disease model fidelity. Having multicellular organoids will allow us to test a more complex pathway interaction that involves multiple cells, such as that which occurs for NASH, alcoholic hepatitis, primary sclerosing cholangitis, etc. (Figure 3).

Our cell-repellent microwell array technology allows for the generation of multicellular clusters, where the composition of each cell type can be precisely controlled to match the one in the real organ (ratio between cells and type of cells constituting the cluster) [26,27]. This technology makes it possible to create organoids from pluripotent stem cells (hESCs or hiPSCs), primary cell lines, and patient-derived cells and hiPSCs [26,27]. 

**Figure 4:**
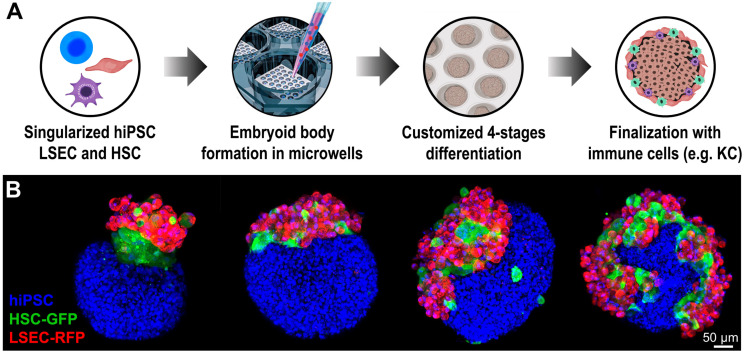
Hybrid strategy for the generation of a multicellular liver organoid model. (**A**) Schematic representation of the different steps to obtain a hybrid multicellular liver organoid model using our agarose micromold technology; (**B**) Immunofluorescence images of our differentiating multicellular liver organoid progression. Differentiating human-induced pluripotent stem cells (hiPSC) labeled with nuclear staining DAPI; Human stellate cells (HSC) labeled with GFP; Liver sinusoidal endothelial cells (LSEC) labeled with RFP. Scale bar 50 μm.

Recently, our group started a project for the generation of multicellular liver organoids where a hybrid model of hiPSC-derived hepatocyte and primary non-parenchymal liver cells (LSEC, HSC) are included in the same liver organoid, intended to be used as liver disease modeling tools (Figure 4A) [156,157]. The importance of having tissue-specific endothelial cells, together with the other non-parenchymal cells in the same ratio found in the liver, will allow us to recreate the native tissue microenvironment that is found in the native tissue, enabling the generation of disease models that could faithfully replicate the disease onset and progression [91,138,144,149,150,151,152,153,154,155]. Our generated multicellular liver organoids showed a polarization of the different cell types within the organoid that was maintained throughout the entire process. (Figure 4B).

## 8. Future Directions

A sustainable source of human liver organoids could impact the treatment of hepatic disorders and the testing of pharmaceutical drugs through the development of reliable disease models. Potential candidates for this application include hepatic progenitor cells from adult or fetal liver, differentiated pluripotent or mesenchymal stem cells, and the direct reprogramming of adult cells. The use of hiPSCs represents an ongoing research field that can potentially obviate the ethical and immune issues related to hESCs. In vitro hepatic functional modeling, in vivo therapy of liver diseases, the testing of novel medications for hepatotoxicity, liver tissue engineering, and the creation of bio-artificial liver (BAL) devices are areas of study and possible treatments utilizing hiPSC-derived liver cells. However, before they can be employed for therapeutic purposes, many steps have to be overcome: (i) enhancing hiPSC generation avoiding viral integration; (ii) preventing the usage of animal components in media for hiPSC culture; (iii) refining differentiation techniques for the improved and more cost-effective development of mature cell types equivalent to primary hepatocytes; (iv) producing quicker protocols for the utilization of patient’s cells for future application; and (v) removing undifferentiated cells that might lead to tumor formation in vivo. A large component of the amelioration of liver differentiation is represented by the addition of supportive cells, such as endothelial cells, which can promote maturation and post-engraftment angiogenesis in vivo. There are no established methodologies for characterizing the morphology, phenotypic, and functional features of differentiated liver organoids. Creating a defined set of morphological and functional metrics for evaluating liver organoids will be an important quality control step to be used in the various models and especially prior to clinical application in the future.

Multicellular organoid generation represents cutting edge technology for future in vitro and in vivo applications, as they can replicate the cellular components that can be found in the native tissue in a most consistent and reproducible way, with the advantage of having a scalable source of liver organoids always available on demand. Liver regenerative therapy represents another potential application for liver organoids, allowing personalized medicine, while avoiding the shortage of organ donors and immune rejection after transplantation.

## Figures and Tables

**Table 1:** List of protocols used for the liver organoid differentiation by several groups.

References	Protocol’s Growth Factors
Agarwal et al. [53]	ActA/FGF4, HGF/BSA, FGF4, HGF/FGF4, HGF, OSM, DEX
Song et al. [54]	ActA /BMP2, FGF4/HGF, KGF/OSM, DEX/OSM, DEX, N2, B27
Touboul et al. [55]	ActA, FGF2/LY-294002, ActA, BMP4, FGF2/FGF10/FGF10, RA, SB431542/FGF4, HGF, EGF
Si-Tayeb et al. [9]	ActA/BMP4, FGF2/HGF/OSM
Vosough et al. [55]	3D differentiation: ActA, Rapa/FGF4, HGF/OSM, DEX
Ogawa et al. [56]	Mix of 3D aggregation and cAMP signaling/Act A, Wnt3a, BMP4/B27, FGF10, FGF2, BMP4/HGF, OSM, DEX
Gieseck et al. [57]	ActA, FGF2, BMP4, LY-294002, CHIR99021/Hepatozyme-SFM, HGF, OSM
Pettinato et al. [8]	3D differentiation: ActA, bFGF, TGFb-1/FGF4, BMP4/DKK-1, WIF-1/OSM, HGF
Pettinato et al. [58]	3D differentiation: ActA, bFGF, TGFb-1, MK-4101/FGF4, BMP4, LY-41575/DKK-1, WIF-1/OSM, HGF, DEX

Acronyms: ActA: Activin A; BMP: Bone Morphogenetic Protein; BSA: Bovine Serum Albumin; DEX: Dexamethasone; DKK−1: Dickkopf−1; FGF: Fibroblast Growth Factor; HGF: Hepatocyte Growth Factor; KGF: Keratinocyte Growth Factor; OSM: Oncostatin M; TGF: Tumor Growth Factor; WIF: Wnt Inhibitory Factor.

**Table 2:** Main features of each cell type and their corresponding 2D and 3D culture systems.

Cell Type	Pros	Cons	2D Culture	3D Culture
Primary hepatocytes isolated from liver	Fully mature and ideal for self-transplantation.	Loss of function in vitro after isolation. Hard to maintain in culture.	Biomatrices, Type IV collagen, laminin, matrigel, soft collagen.	Fiber bonding, freeze drying, gas foaming, melt molding, particulate leaching, and phase separation.
Human embryonic stem cells (hESCs)	Pluripotent capabilities to obtain any type of cells.	Ethical debates, possible generation of teratomas.	Biomatrices, collagen, matrigel, vitronectin.	Biodegradable polymers, hollow fiber, rotating bioreactor, 3D spheroid culture systems.
Human-induced pluripotent stem cells (hiPSCs)	Exclude ethical debates, patient’s autologous generation prevent immune suppression/rejection.	Epigenetic memory that might impair differentiation abilities.	Biomatrices, collagen, matrigel, vitronectin.	Hollow fiber/organoids, micro-cavitary hydrogel (MCG) system, Swiss 3T3 cell sheets, 3D spheroid culture systems.
Hepatic progenitor cells	Able to differentiate into mature hepatocyte.	Challenging to isolate.	Type IV collagen or laminin.	Bioartificial liver systems, biomatrix scaffolds, fibroblast feeder layers, nanofiber and alginate scaffolds, 3D collagen gel matrix, 3D matrixes of poly (ethylene glycol)-bpoly-(L-alanine) thermogel.
Mesenchymal stem cells: Adipose tissue, bone marrow, placental cells, umbilical cord amniotic cells.	Possibility to be isolated from the same patient avoiding immune rejection	Difficult to differentiate because of epigenetic memory and need of an initial dedifferentiation step.	Biomatrices, Type IV collagen, laminin, matrigel, soft collagen	Bioartificial liver systems, nanofibers and alginate scaffolds 3D matrixes of poly (ethylene glycol)-b-poly-(L-alanine) thermogel.

## Data Availability

Not applicable.

## References

[1] Alqahtani S., Larson A.M. (2011). Adult liver transplantation in the USA. Curr. Opin. Gastroenterol..

[2] Dianat N., Steichen C., Vallier L., Weber A., Dubart-Kupperschmitt A. (2013). Human Pluripotent Stem Cells for Modelling Human Liver Diseases and Cell Therapy. Curr. Gene Ther..

[3] Terry C., Dhawan A., Mitry R.R., Lehec S.C., Hughes R.D. (2010). Optimization of the cryopreservation and thawing protocol for human hepatocytes for use in cell transplantation. Liver Transplant..

[4] Aasen T., Raya A., Barrero M., Garreta E., Consiglio A., Gonzalez F., Vassena R., Bilić J., Pekarik V., Tiscornia G. (2008). Efficient and rapid generation of induced pluripotent stem cells from human keratinocytes. Nat. Biotechnol..

[5] Takahashi K., Tanabe K., Ohnuki M., Narita M., Ichisaka T., Tomoda K., Yamanaka S. (2007). Induction of Pluripotent Stem Cells from Adult Human Fibroblasts by Defined Factors. Cell.

[6] Yu J., Vodyanik M.A., Smuga-Otto K., Antosiewicz-Bourget J., Frane J.L., Tian S., Nie J., Jonsdottir G.A., Ruotti V., Stewart R. (2007). Induced Pluripotent Stem Cell Lines Derived from Human Somatic Cells. Science.

[7] Raju R., Chau D., Verfaillie C.M., Hu W.-S. (2013). The road to regenerative liver therapies: The triumphs, trials and tribulations. Biotechnol. Adv..

[8] Pettinato G., Ramanathan R., Fisher R.A., Mangino M.J., Zhang N., Wen X. (2016). Scalable Differentiation of Human iPSCs in a Multicellular Spheroid-based 3D Culture into Hepatocyte-like Cells through Direct Wnt/β-catenin Pathway Inhibition. Sci. Rep..

[9] Si-Tayeb K., Noto F.K., Nagaoka M., Li J., Battle M.A., Duris C., North P.E., Dalton S., Duncan S.A. (2010). Highly efficient generation of human hepatocyte-like cells from induced pluripotent stem cells. Hepatology.

[10] Zhu X., Zhang B., He Y., Bao J. (2021). Liver Organoids: Formation Strategies and Biomedical Applications. Tissue Eng. Regen. Med..

[11] De Rudder M., Dili A., Stärkel P., Leclercq I.A. (2021). Critical Role of LSEC in Post-Hepatectomy Liver Regeneration and Failure. Int. J. Mol. Sci..

[12] Kosmacheva S.M., Seviaryn I.N., Goncharova N.V., Petyovka N.V., Potapnev M.P. (2011). Hepatogenic Potential of Human Bone Marrow and Umbilical Cord Blood Mesenchymal Stem Cells. Bull. Exp. Biol. Med..

[13] Wu X.-B., Tao R. (2012). Hepatocyte differentiation of mesenchymal stem cells. Hepatobiliary Pancreat. Dis. Int..

[14] Hannan N., Segeritz C.-P., Touboul T., Vallier L. (2013). Production of hepatocyte-like cells from human pluripotent stem cells. Nat. Protoc..

[15] Heslop J.A., Rowe C., Walsh J., Sison-Young R., Jenkins R., Kamalian L., Kia R., Hay D., Jones R.P., Malik H.Z. (2017). Mechanistic evaluation of primary human hepatocyte culture using global proteomic analysis reveals a selective dedifferentiation profile. Arch. Toxicol..

[16] Tong J.Z., De Lagausie P., Furlan V., Cresteil T., Bernard O., Alvarez F. (1992). Long-term culture of adult rat hepatocyte spheroids. Exp. Cell Res..

[17] Höpfl G., Gassmann M., Desbaillets I., Schatten H. (2004). Differentiating Embryonic Stem Cells into Embryoid Bodies. Germ Cell Protocols.

[18] Khoo M.L., McQuade L.R., Smith M.S., Lees J.G., Sidhu K.S., Tuch B.E. (2005). Growth and Differentiation of Embryoid Bodies Derived from Human Embryonic Stem Cells: Effect of Glucose and Basic Fibroblast Growth Factor. Biol. Reprod..

[19] Mohr J.C., Zhang J., Azarin S.M., Soerens A.G., de Pablo J.J., Thomson J.A., Lyons G.E., Palecek S.P., Kamp T.J. (2010). The microwell control of embryoid body size in order to regulate cardiac differentiation of human embryonic stem cells. Biomaterials.

[20] Messana J.M., Hwang N.S., Coburn J., Elisseeff J.H., Zhang Z. (2008). Size of the embryoid body influences chondrogenesis of mouse embryonic stem cells. J. Tissue Eng. Regen. Med..

[21] Van Winkle A.P., Gates I.D., Kallos M.S. (2012). Mass Transfer Limitations in Embryoid Bodies during Human Embryonic Stem Cell Differentiation. Cell. Tissues Organs.

[22] Friedrich J., Seidel C., Ebner R., Kunz-Schughart L.A. (2009). Spheroid-based drug screen: Considerations and practical approach. Nat. Protoc..

[23] Kelm J.M., Timmins N.E., Brown C.J., Fussenegger M., Nielsen L.K. (2003). Method for generation of homogeneous multicellular tumor spheroids applicable to a wide variety of cell types. Biotechnol. Bioeng..

[24] Chang T.T., Hughes-Fulford M. (2009). Monolayer and Spheroid Culture of Human Liver Hepatocellular Carcinoma Cell Line Cells Demonstrate Distinct Global Gene Expression Patterns and Functional Phenotypes. Tissue Eng. Part A.

[25] Fukuda J., Sakai Y., Nakazawa K. (2006). Novel hepatocyte culture system developed using microfabrication and collagen/polyethylene glycol microcontact printing. Biomaterials.

[26] Pettinato G., Wen X., Zhang N. (2014). Formation of Well-defined Embryoid Bodies from Dissociated Human Induced Pluripotent Stem Cells using Microfabricated Cell-repellent Microwell Arrays. Sci. Rep..

[27] Pettinato G., Berg-Foels W.S.V., Zhang N., Wen X. (2014). ROCK Inhibitor Is Not Required for Embryoid Body Formation from Singularized Human Embryonic Stem Cells. PLoS ONE.

[28] Pettinato G., Wen X., Zhang N. (2015). Engineering Strategies for the Formation of Embryoid Bodies from Human Pluripotent Stem Cells. Stem Cell. Dev..

[29] Joannides A.J., Fiore-Hériché C., Battersby A.A., Athauda-Arachchi P., Bouhon I.A., Williams L., Westmore K., Kemp P.J., Compston A., Allen N.D. (2007). A Scaleable and Defined System for Generating Neural Stem Cells from Human Embryonic Stem Cells. Stem Cell..

[30] Lock L.T., Tzanakakis E.S. (2009). Expansion and Differentiation of Human Embryonic Stem Cells to Endoderm Progeny in a Microcarrier Stirred-Suspension Culture. Tissue Eng. Part A.

[31] Nieden N.I.Z., Cormier J.T., Rancourt D.E., Kallos M.S. (2007). Embryonic stem cells remain highly pluripotent following long term expansion as aggregates in suspension bioreactors. J. Biotechnol..

[32] Taiani J.T., Krawetz R.J., Nieden N.I.Z., Wu Y.E., Kallos M.S., Matyas J.R., Rancourt D.E. (2010). Reduced Differentiation Efficiency of Murine Embryonic Stem Cells in Stirred Suspension Bioreactors. Stem Cell. Dev..

[33] Watanabe K., Ueno M., Kamiya D., Nishiyama A., Matsumura M., Wataya T., Takahashi J.B., Nishikawa S., Nishikawa S.-I., Muguruma K. (2007). A ROCK inhibitor permits survival of dissociated human embryonic stem cells. Nat. Biotechnol..

[34] Chaddah R., Arntfield M., Runciman S., Clarke L., Van Der Kooy D. (2012). Clonal Neural Stem Cells from Human Embryonic Stem Cell Colonies. J. Neurosci..

[35] Ng E.S., Davis R., Azzola L., Stanley E.G., Elefanty A. (2005). Forced aggregation of defined numbers of human embryonic stem cells into embryoid bodies fosters robust, reproducible hematopoietic differentiation. Blood.

[36] Sheridan S.D., Surampudi V., Rao R.R. (2012). Analysis of Embryoid Bodies Derived from Human Induced Pluripotent Stem Cells as a Means to Assess Pluripotency. Stem Cell. Int..

[37] Higgins G., Anderson R., Higgins G., Anderson R. (1931). Experimental pathology of the liver: Restoration of the liver of the white rat following partial surgical removal. Arch. Pathol..

[38] Yanger K., Knigin D., Zong Y., Maggs L., Gu G., Akiyama H., Pikarsky E., Stanger B.Z. (2014). Adult Hepatocytes Are Generated by Self-Duplication Rather than Stem Cell Differentiation. Cell Stem Cell.

[39] Wei Y., Wang Y.G., Jia Y., Li L., Yoon J., Zhang S., Wang Z., Zhang Y., Zhu M., Sharma T. (2021). Liver homeostasis is maintained by midlobular zone 2 hepatocytes. Science.

[40] Tsomaia K., Patarashvili L., Karumidze N., Bebiashvili I., Az-Maipharashvili E., Modebadze I., Dzidziguri D., Sareli M., Gusev S., Kordzaia D. (2020). Liver structural transformation after partial hepatectomy and repeated partial hepatectomy in rats: A renewed view on liver regeneration. World J. Gastroenterol..

[41] Kholodenko I.V., Yarygin K.N. (2017). Cellular Mechanisms of Liver Regeneration and Cell-Based Therapies of Liver Diseases. BioMed Res. Int..

[42] Sato Y., Koyama S., Tsukada K., Hatakeyama K. (1997). Acute portal hypertension reflecting shear stress as a trigger of liver regeneration following partial hepatectomy. Surg. Today.

[43] Lory J., Schweizer W., Blumgart L.H., Zimmermann A. (1994). The pathology of the atrophy/hypertrophy complex (AHC) of the liver. A light microscopic and immunohistochemical study. Histol. Histopathol..

[44] García I.C., Villalba J.S., Iovino D., Franchi C., Iori V., Pettinato G., Inversini D., Amico F., Ietto G. (2022). Liver Trauma: Until When We Have to Delay Surgery? A Review. Life.

[45] Poisson J., Lemoinne S., Boulanger C., Durand F., Moreau R., Valla D., Rautou P.-E. (2017). Liver sinusoidal endothelial cells: Physiology and role in liver diseases. J. Hepatol..

[46] Currier A.R., Sabla G., Locaputo S., Melin-Aldana H., Degen J.L., Bezerra J.A. (2003). Plasminogen directs the pleiotropic effects of uPA in liver injury and repair. Am. J. Physiol. Liver Physiol..

[47] Fisher R.A., Strom S. (2006). Human Hepatocyte Transplantation: Worldwide Results. Transplantation.

[48] Forbes S.J., Gupta S., Dhawan A. (2015). Cell therapy for liver disease: From liver transplantation to cell factory. J. Hepatol..

[49] Kadyk L.C., Collins L.R., Littman N.J., Millan M.T. (2015). Proceedings: Moving Toward Cell-Based Therapies for Liver Disease. Stem Cell. Transl. Med..

[50] Nicolas C.T., Wang Y., Nyberg S.L. (2016). Cell therapy in chronic liver disease. Curr. Opin. Gastroenterol..

[51] Yu Y., Wang X., Nyberg S.L. (2014). Potential and Challenges of Induced Pluripotent Stem Cells in Liver Diseases Treatment. J. Clin. Med..

[52] Singh V.K., Kalsan M., Kumar N., Saini A., Chandra R. (2015). Induced pluripotent stem cells: Applications in regenerative medicine, disease modeling, and drug discovery. Front. Cell Dev. Biol..

[53] D’Amour K.A., Agulnick A.D., Eliazer S., Kelly O.G., Kroon E., Baetge E.E. (2005). Efficient differentiation of human embryonic stem cells to definitive endoderm. Nat. Biotechnol..

[54] Song Z., Cai J., Liu Y., Zhao D., Yong J., Duo S., Song X., Guo Y., Zhao Y., Qin H. (2009). Efficient generation of hepatocyte-like cells from human induced pluripotent stem cells. Cell Res..

[55] Touboul T., Hannan N.R.F., Corbineau S., Martinez A., Martinet C., Branchereau S., Mainot S., Strick-Marchand H., Pedersen R., Di Santo J. (2010). Generation of functional hepatocytes from human embryonic stem cells under chemically defined conditions that recapitulate liver development. Hepatology.

[56] Ogawa S., Surapisitchat J., Virtanen C., Ogawa M., Niapour M., Sugamori K.S., Wang S., Tamblyn L., Guillemette C., Hoffmann E. (2013). Three-dimensional culture and cAMP signaling promote the maturation of human pluripotent stem cell-derived hepatocytes. Development.

[57] Iii R.L.G., Hannan N., Bort R., Hanley N., Drake R.A.L., Cameron G.W.W., Wynn T., Vallier L. (2014). Maturation of Induced Pluripotent Stem Cell Derived Hepatocytes by 3D-Culture. PLoS ONE.

[58] Pettinato G., Coughlan M.F., Zhang X., Chen L., Khan U., Glyavina M., Sheil C.J., Upputuri P.K., Zakharov Y.N., Vitkin E. (2021). Spectroscopic label-free microscopy of changes in live cell chromatin and biochemical composition in transplantable organoids. Sci. Adv..

[59] Duncan S.A. (2000). Transcriptional regulation of liver development. Dev. Dyn. Off. Publ. Am. Assoc. Anat..

[60] Duboc V., Lapraz F., Saudemont A., Bessodes N., Mekpoh F., Haillot E., Quirin M., Lepage T. (2010). Nodal and BMP2/4 pattern the mesoderm and endoderm during development of the sea urchin embryo. Development.

[61] Morrison G.M., Oikonomopoulou I., Migueles R.P., Soneji S., Livigni A., Enver T., Brickman J.M. (2008). Anterior Definitive Endoderm from ESCs Reveals a Role for FGF Signaling. Cell Stem Cell.

[62] Gadue P., Huber T.L., Paddison P.J., Keller G.M. (2006). Wnt and TGF-beta signaling are required for the induction of an in vitro model of primitive streak formation using embryonic stem cells. Proc. Natl. Acad. Sci. USA.

[63] Agarwal S., Holton K.L., Lanza R. (2008). Efficient Differentiation of Functional Hepatocytes from Human Embryonic Stem Cells. Stem Cell..

[64] Jung J., Zheng M., Goldfarb M., Zaret K.S. (1999). Initiation of Mammalian Liver Development from Endoderm by Fibroblast Growth Factors. Science.

[65] Rossi J.M., Dunn N.R., Hogan B.L., Zaret K.S. (2001). Distinct mesodermal signals, including BMPs from the septum transversum mesenchyme, are required in combination for hepatogenesis from the endoderm. Genes Dev..

[66] Kamiya A., Kinoshita T., Ito Y., Matsui T., Morikawa Y., Senba E., Nakashima K., Taga T., Yoshida K., Kishimoto T. (1999). Fetal liver development requires a paracrine action of oncostatin M through the gp130 signal transducer. EMBO J..

[67] Huang P., He Z., Ji S., Sun H., Xiang D., Liu C., Hu Y., Wang X., Hui L. (2011). Induction of functional hepatocyte-like cells from mouse fibroblasts by defined factors. Nature.

[68] Nakamori D., Takayama K., Nagamoto Y., Mitani S., Sakurai F., Tachibana M., Mizuguchi H. (2016). Hepatic maturation of human iPS cell-derived hepatocyte-like cells by ATF5, c/EBPα, and PROX1 transduction. Biochem. Biophys. Res. Commun..

[69] Snykers S., De Kock J., Rogiers V., Vanhaecke T. (2009). In Vitro Differentiation of Embryonic and Adult Stem Cells into Hepatocytes: State of the Art. Stem Cell..

[70] Cadigan K.M., Nusse R. (1997). Wnt signaling: A common theme in animal development. Genes Dev..

[71] Hoeflich K.P., Luo J., Rubie E.A., Tsao M.-S., Jin O., Woodgett J.R. (2000). Requirement for glycogen synthase kinase-3β in cell survival and NF-κB activation. Nature.

[72] Monga S., Monga H.K., Tan X., Mulé K., Pediaditakis P., Michalopoulos G.K. (2003). β-catenin antisense studies in embryonic liver cultures: Role in proliferation, apoptosis, and lineage specification. Gastroenterology.

[73] Nejak-Bowen K., Monga S.P. (2008). Wnt/β-catenin signaling in hepatic organogenesis. Organogenesis.

[74] McLin V.A., Rankin S.A., Zorn A.M. (2007). Repression of Wnt/β-catenin signaling in the anterior endoderm is essential for liver and pancreas development. Development.

[75] Decaens T., Godard C., de Reyniès A., Rickman D.S., Tronche F., Couty J.-P., Perret C., Colnot S. (2008). Stabilization of β-catenin affects mouse embryonic liver growth and hepatoblast fate. Hepatology.

[76] So J., Martin B., Kimelman D., Shin D. (2013). Wnt/β-catenin signaling cell-autonomously converts non-hepatic endodermal cells to a liver fate. Biol. Open.

[77] Sekiya S., Suzuki A. (2011). Direct conversion of mouse fibroblasts to hepatocyte-like cells by defined factors. Nature.

[78] Yahoo N., Pournasr B., Rostamzadeh J., Hakhamaneshi M.S., Ebadifar A., Fathi F., Baharvand H. (2016). Forced expression of Hnf1b/Foxa3 promotes hepatic fate of embryonic stem cells. Biochem. Biophys. Res. Commun..

[79] Yahoo N., Pournasr B., Rostamzadeh J., Fathi F. (2016). Forced expression of Hnf4a induces hepatic gene activation through directed differentiation. Biochem. Biophys. Res. Commun..

[80] Cao H., Yang J., Yu J., Pan Q., Li J., Zhou P., Li Y., Pan X., Li J., Wang Y. (2012). Therapeutic potential of transplanted placental mesenchymal stem cells in treating Chinese miniature pigs with acute liver failure. BMC Med..

[81] Hu X., Xie P., Li W., Li Z., Shan H. (2016). Direct induction of hepatocyte-like cells from immortalized human bone marrow mesenchymal stem cells by overexpression of HNF4α. Biochem. Biophys. Res. Commun..

[82] Bao J., Wu Q., Wang Y., Li Y., Li L., Chen F., Wu X., Xie M., Bu H. (2016). Enhanced hepatic differentiation of rat bone marrow-derived mesenchymal stem cells in spheroidal aggregate culture on a decellularized liver scaffold. Int. J. Mol. Med..

[83] Kwon M.-J., Kang S.-J., Park Y.-I., Yang Y.-H., Bang S.-I., Park Y.H., So B., Cho M.-H., Kang H.-G. (2015). Hepatic differentiation of human adipose tissue-derived mesenchymal stem cells and adverse effects of arsanilic acid and acetaminophen during in vitro hepatic developmental stage. Cell Biol. Toxicol..

[84] Zhu X.-Q., Pan X.-H., Yao L., Li W., Cui J., Wang G., Mrsny R.J., Hoffman A.R., Hu J.-F. (2016). Converting Skin Fibroblasts into Hepatic-like Cells by Transient Programming. J. Cell. Biochem..

[85] De Kock J., Rodrigues R.M., Buyl K., Vanhaecke T., Rogiers V. (2015). Human Skin-Derived Precursor Cells: Isolation, Expansion, and Hepatic Differentiation. Methods Mol. Biol..

[86] Chen Z., Kuang Q., Lao X.-J., Yang J., Huang W., Zhou D. (2016). Differentiation of UC-MSCs into hepatocyte-like cells in partially hepatectomized model rats. Exp. Ther. Med..

[87] Raut A., Khanna A. (2017). High-throughput sequencing to identify microRNA signatures during hepatic differentiation of human umbilical cord Wharton’s jelly-derived mesenchymal stem cells. Hepatol. Res..

[88] Ramanathan R., Pettinato G., Beeston J.T., Lee D.D., Wen X., Mangino M.J., Fisher R.A. (2015). Transplantation of human stem cell-derived hepatocytes in an animal model of acute liver failure. Surgery.

[89] Chen K.G., Mallon B.S., McKay R.D.G., Robey P.G. (2014). Human Pluripotent Stem Cell Culture: Considerations for Maintenance, Expansion, and Therapeutics. Cell Stem Cell.

[90] Iwasawa K., Takebe T. (2021). Organogenesis in vitro. Curr. Opin. Cell Biol..

[91] Thompson W.L., Takebe T. (2021). Human liver model systems in a dish. Dev. Growth Differ..

[92] Thompson W.L., Takebe T. (2020). Generation of multi-cellular human liver organoids from pluripotent stem cells. Methods Cell Biol..

[93] Dasgupta A., Hughey R., Lancin P., Larue L., Moghe P.V. (2005). E-cadherin synergistically induces hepatospecific phenotype and maturation of embryonic stem cells in conjunction with hepatotrophic factors. Biotechnol. Bioeng..

[94] Frith J.E., Thomson B., Genever P.G. (2010). Dynamic Three-Dimensional Culture Methods Enhance Mesenchymal Stem Cell Properties and Increase Therapeutic Potential. Tissue Eng. Part C Methods.

[95] Vosough M., Omidinia E., Kadivar M., Shokrgozar M.-A., Pournasr B., Aghdami N., Baharvand H. (2013). Generation of Functional Hepatocyte-Like Cells from Human Pluripotent Stem Cells in a Scalable Suspension Culture. Stem Cell. Dev..

[96] Glicklis R., Shapiro L., Agbaria R., Merchuk J.C., Cohen S. (2000). Hepatocyte behavior within three-dimensional porous alginate scaffolds. Biotechnol. Bioeng..

[97] Ramaiahgari S.C., den Braver M.W., Herpers B., Terpstra V., Commandeur J.N.M., Van De Water B., Price L.S. (2014). A 3D in vitro model of differentiated HepG2 cell spheroids with improved liver-like properties for repeated dose high-throughput toxicity studies. Arch. Toxicol..

[98] Sivertsson L., Synnergren J., Jensen J., Björquist P., Ingelman-Sundberg M. (2013). Hepatic Differentiation and Maturation of Human Embryonic Stem Cells Cultured in a Perfused Three-Dimensional Bioreactor. Stem Cell. Dev..

[99] Ramasamy T.S., Yu J.S., Selden A., Hodgson H., Cui W. (2013). Application of Three-Dimensional Culture Conditions to Human Embryonic Stem Cell-Derived Definitive Endoderm Cells Enhances Hepatocyte Differentiation and Functionality. Tissue Eng. Part A.

[100] Miki T., Ring A., Gerlach J. (2011). Hepatic Differentiation of Human Embryonic Stem Cells Is Promoted by Three-Dimensional Dynamic Perfusion Culture Conditions. Tissue Eng. Part C Methods.

[101] Khetani S.R., Bhatia S.N. (2008). Microscale culture of human liver cells for drug development. Nat. Biotechnol..

[102] Subramanian K., Owens D.J., Raju R., Firpo M., O’Brien T., Verfaillie C.M., Hu W.-S. (2014). Spheroid Culture for Enhanced Differentiation of Human Embryonic Stem Cells to Hepatocyte-Like Cells. Stem Cell. Dev..

[103] An S.Y., Woo D.-H., Han J., Kim J.H., Jang Y.J., Son J.S., Yang H., Cheon Y.P., Wang M., Zhang P. (2013). Engraftment Potential of Spheroid-Forming Hepatic Endoderm Derived from Human Embryonic Stem Cells. Stem Cell. Dev..

[104] Zhang R.-R., Takebe T., Miyazaki L., Takayama M., Koike H., Kimura M., Enomura M., Zheng Y.-W., Sekine K., Taniguchi H. (2014). Efficient Hepatic Differentiation of Human Induced Pluripotent Stem Cells in a Three-Dimensional Microscale Culture. Methods Mol. Biol..

[105] Takayama K., Kawabata K., Nagamoto Y., Kishimoto K., Tashiro K., Sakurai F., Tachibana M., Kanda K., Hayakawa T., Furue M.K. (2013). 3D spheroid culture of hESC/hiPSC-derived hepatocyte-like cells for drug toxicity testing. Biomaterials.

[106] Stevens K.R., Ungrin M.D., Schwartz R.E., Ng S., Carvalho B., Christine K.S., Chaturvedi R.R., Li C.Y., Zandstra P.W., Chen C.S. (2013). InVERT molding for scalable control of tissue microarchitecture. Nat. Commun..

[107] Gothard D., Roberts S.J., Shakesheff K.M., Buttery L.D. (2009). Controlled embryoid body formation via surface modification and avidin–biotin cross-linking. Cytotechnology.

[108] Buttery L.D.K., Bourne S., Xynos J.D., Wood H., Hughes F.J., Hughes S.P.F., Episkopou V., Polak J.M. (2001). Differentiation of Osteoblasts and in Vitro Bone Formation from Murine Embryonic Stem Cells. Tissue Eng..

[109] Yirme G., Amit M., Laevsky I., Osenberg S., Itskovitz-Eldor J. (2008). Establishing a Dynamic Process for the Formation, Propagation, and Differentiation of Human Embryoid Bodies. Stem Cell. Dev..

[110] Feraud O., Cao Y., Vittet D. (2001). Embryonic Stem Cell-Derived Embryoid Bodies Development in Collagen Gels Recapitulates Sprouting Angiogenesis. Lab. Investig..

[111] Pal R., Mamidi M.K., Das A.K., Gupta P.K., Bhonde R. (2012). A Simple and economical route to generate functional hepatocyte-like cells from hESCs and their application in evaluating alcohol induced liver damage. J. Cell. Biochem..

[112] Berger D.R., Ware B.R., Davidson M.D., Allsup S.R., Khetani S.R. (2015). Enhancing the functional maturity of induced pluripotent stem cell–derived human hepatocytes by controlled presentation of cell–cell interactions in vitro. Hepatology.

[113] Soto-Gutiérrez A., Navarro-Alvarez N., Zhao D., Rivascarrillo J.D., Lebkowski J., Tanaka N., Fox I.J., Kobayashi N. (2007). Differentiation of mouse embryonic stem cells to hepatocyte-like cells by co-culture with human liver nonparenchymal cell lines. Nat. Protoc..

[114] Han S., Dziedzic N., Gadue P., Keller G.M., Gouon-Evans V. (2011). An Endothelial Cell Niche Induces Hepatic Specification Through Dual Repression of Wnt and Notch Signaling. Stem Cell..

[115] Takebe T., Zhang R., Koike H., Kimura M., Yoshizawa E., Enomura M., Koike N., Sekine K., Taniguchi H. (2014). Generation of a vascularized and functional human liver from an iPSC-derived organ bud transplant. Nat. Protoc..

[116] Zhong L., Gou J., Deng N., Shen H., He T., Zhang B.-Q. (2015). Three-dimensional Co-culture of hepatic progenitor cells and mesenchymal stem cells in vitro and in vivo. Microsc. Res. Tech..

[117] Mizumoto H., Aoki K., Nakazawa K., Ijima H., Funatsu K., Kajiwara T. (2008). Hepatic Differentiation of Embryonic Stem Cells in HF/Organoid Culture. Transplant. Proc..

[118] Lee J.Y., Tuleuova N., Jones C.N., Ramanculov E., Zern M.A., Revzin A. (2009). Directing hepatic differentiation of embryonic stem cells with protein microarray-based co-cultures. Integr. Biol..

[119] Li L., Sharma N., Chippada U., Jiang X., Schloss R., Yarmush M.L., Langrana N.A. (2008). Functional Modulation of ES-Derived Hepatocyte Lineage Cells via Substrate Compliance Alteration. Ann. Biomed. Eng..

[120] Amimoto N., Mizumoto H., Nakazawa K., Ijima H., Funatsu K., Kajiwara T. (2011). Hepatic Differentiation of Mouse Embryonic Stem Cells and Induced Pluripotent Stem Cells During Organoid Formation in Hollow Fibers. Tissue Eng. Part A.

[121] Liu T., Zhang S., Chen X., Li G., Wang Y. (2010). Hepatic Differentiation of Mouse Embryonic Stem Cells in Three-Dimensional Polymer Scaffolds. Tissue Eng. Part A.

[122] Farzaneh Z., Pournasr B., Ebrahimi M., Aghdami N., Baharvand H. (2010). Enhanced Functions of Human Embryonic Stem Cell-derived Hepatocyte-like Cells on Three-dimensional Nanofibrillar Surfaces. Stem Cell Rev. Rep..

[123] Fang S., Qiu Y.-D., Mao L., Shi X.-L., Yu D.-C., Ding Y.-T. (2007). Differentiation of embryoid-body cells derived from embryonic stem cells into hepatocytes in alginate microbeads in vitro. Acta Pharmacol. Sin..

[124] Malinen M.M., Kanninen L.K., Corlu A., Isoniemi H.M., Lou Y.-R., Yliperttula M.L., Urtti A.O. (2014). Differentiation of liver progenitor cell line to functional organotypic cultures in 3D nanofibrillar cellulose and hyaluronan-gelatin hydrogels. Biomaterials.

[125] Haque A., Hexig B., Meng Q., Hossain S., Nagaoka M., Akaike T. (2011). The effect of recombinant E-cadherin substratum on the differentiation of endoderm-derived hepatocyte-like cells from embryonic stem cells. Biomaterials.

[126] Kanninen L.K., Porola P., Niklander J., Malinen M.M., Corlu A., Guguen-Guillouzo C., Urtti A., Yliperttula M.L., Lou Y.-R. (2016). Hepatic differentiation of human pluripotent stem cells on human liver progenitor HepaRG-derived acellular matrix. Exp. Cell Res..

[127] Kanninen L.K., Harjumäki R., Peltoniemi P., Bogacheva M.S., Salmi T., Porola P., Niklander J., Smutný T., Urtti A., Yliperttula M.L. (2016). Laminin-511 and laminin-521-based matrices for efficient hepatic specification of human pluripotent stem cells. Biomaterials.

[128] Michielin F., Giobbe G.G., Luni C., Hu Q., Maroni I., Orford M.R., Manfredi A., Di Filippo L., David A.L., Cacchiarelli D. (2020). The Microfluidic Environment Reveals a Hidden Role of Self-Organizing Extracellular Matrix in Hepatic Commitment and Organoid Formation of hiPSCs. Cell Rep..

[129] Bao J., Shi Y., Sun H., Yin X., Yang R., Li L., Chen X., Bu H. (2011). Construction of a Portal Implantable Functional Tissue-Engineered Liver Using Perfusion-Decellularized Matrix and Hepatocytes in Rats. Cell Transplant..

[130] Skardal A., Smith L., Bharadwaj S., Atala A., Soker S., Zhang Y. (2012). Tissue specific synthetic ECM hydrogels for 3-D in vitro maintenance of hepatocyte function. Biomaterials.

[131] Geerts S., Ozer S., Jaramillo M., Yarmush M.L., Uygun B.E. (2016). Nondestructive Methods for Monitoring Cell Removal During Rat Liver Decellularization. Tissue Eng. Part C Methods.

[132] Mazza G., Rombouts K., Hall A.R., Urbani L., Luong T.V., Al-Akkad W., Longato L., Brown D., Maghsoudlou P., Dhillon A.P. (2015). Decellularized human liver as a natural 3D-scaffold for liver bioengineering and transplantation. Sci. Rep..

[133] Shahbazi M.N. (2020). Mechanisms of human embryo development: From cell fate to tissue shape and back. Development.

[134] Harrison S.E., Sozen B., Christodoulou N., Kyprianou C., Zernicka-Goetz M. (2017). Assembly of embryonic and extraembryonic stem cells to mimic embryogenesis in vitro. Science.

[135] Al Reza H., Okabe R., Takebe T. (2021). Organoid transplant approaches for the liver. Transpl. Int..

[136] Tirziu D., Simons M. (2009). Endothelium as master regulator of organ development and growth. Vasc. Pharmacol..

[137] Belle M., Godefroy D., Couly G., Malone S.A., Collier F., Giacobini P., Chédotal A. (2017). Tridimensional Visualization and Analysis of Early Human Development. Cell.

[138] Matsumoto K., Yoshitomi H., Rossant J., Zaret K.S. (2001). Liver Organogenesis Promoted by Endothelial Cells Prior to Vascular Function. Science.

[139] Rafii S., Butler J.M., Ding B.-S. (2016). Angiocrine functions of organ-specific endothelial cells. Nature.

[140] Butler J.M., Kobayashi H., Rafii S. (2010). Instructive role of the vascular niche in promoting tumour growth and tissue repair by angiocrine factors. Nat. Rev. Cancer.

[141] Nolan D.J., Ginsberg M., Israely E., Palikuqi B., Poulos M.G., James D., Ding B.-S., Schachterle W., Liu Y., Rosenwaks Z. (2013). Molecular Signatures of Tissue-Specific Microvascular Endothelial Cell Heterogeneity in Organ Maintenance and Regeneration. Dev. Cell.

[142] Takebe T., Sekine K., Enomura M., Koike H., Kimura M., Ogaeri T., Zhang R., Ueno Y., Zheng Y.-W., Koike N. (2013). Vascularized and functional human liver from an iPSC-derived organ bud transplant. Nature.

[143] Wang B., Zhao L., Fish M., Logan C.Y., Nusse R. (2015). Self-renewing diploid Axin2+ cells fuel homeostatic renewal of the liver. Nature.

[144] Michalopoulos G.K., Grompe M., Theise N.D. (2013). Assessing the potential of induced liver regeneration. Nat. Med..

[145] Pettinato G., Lehoux S., Ramanathan R., Salem M.M., He L.-X., Muse O., Flaumenhaft R., Thompson M.T., Rouse E.A., Cummings R.D. (2019). Generation of fully functional hepatocyte-like organoids from human induced pluripotent stem cells mixed with Endothelial Cells. Sci. Rep..

[146] Pettinato G. (2022). Generation of Hepatocyte Organoids from Human iPS Cells. Hepatocytes: Methods and Protocols.

[147] Sun X., Aghazadeh Y., Nunes S.S. (2022). Isolation of ready-made rat microvessels and its applications in effective in vivo vascularization and in angiogenic studies in vitro. Nat. Protoc..

[148] Panwar A., Das P., Tan L.P. (2021). 3D Hepatic Organoid-Based Advancements in LIVER Tissue Engineering. Bioengineering.

[149] Lee H., Mun S.J., Jung C., Kang H., Kwon J., Ryu J., Ahn H., Kwon O., Ahn J., Moon K. (2023). In vitro modeling of liver fibrosis with 3D co-culture system using a novel human hepatic stellate cell line. Biotechnol. Bioeng..

[150] Pastore M., Caligiuri A., Raggi C., Navari N., Piombanti B., Di Maira G., Rovida E., Piccinni M.-P., Lombardelli L., Logiodice F. (2022). Macrophage MerTK promotes profibrogenic cross-talk with hepatic stellate cells via soluble mediators. JHEP Rep..

[151] Nam D., Park M.R., Lee H., Bae S.C., Gerovska D., Araúzo-Bravo M.J., Zaehres H., Schöler H.R., Kim J.B. (2022). Induced Endothelial Cell-Integrated Liver Assembloids Promote Hepatic Maturation and Therapeutic Effect on Cholestatic Liver Fibrosis. Cells.

[152] Guo Q., Furuta K., Islam S., Caporarello N., Kostallari E., Dielis K., Tschumperlin D.J., Hirsova P., Ibrahim S.H. (2022). Liver sinusoidal endothelial cell expressed vascular cell adhesion molecule 1 promotes liver fibrosis. Front. Immunol..

[153] Cao D., Ge J.-Y., Wang Y., Oda T., Zheng Y.-W. (2021). Hepatitis B virus infection modeling using multi-cellular organoids derived from human induced pluripotent stem cells. World J. Gastroenterol..

[154] Brovold M., Keller D., Soker S. (2020). Differential fibrotic phenotypes of hepatic stellate cells within 3D liver organoids. Biotechnol. Bioeng..

[155] Ouchi R., Togo S., Kimura M., Shinozawa T., Koido M., Koike H., Thompson W., Karns R.A., Mayhew C., McGrath P.S. (2019). Modeling Steatohepatitis in Humans with Pluripotent Stem Cell-Derived Organoids. Cell Metab..

[156] Matsuzaki T., Shimokawa Y., Koike H., Kimura M., Kawano Y., Okuma N., Kawamura R., Yoneyama Y., Furuichi Y., Hakuno F. (2022). Mechanical guidance of self-condensation patterns of differentiating progeny. iScience.

[157] Kim H.J., Kim G., Chi K.Y., Kim H., Jang Y.J., Jo S., Lee J., Lee Y., Woo D.-H., Han C. (2023). Generation of multilineage liver organoids with luminal vasculature and bile ducts from human pluripotent stem cells via modulation of Notch signaling. Stem Cell Res. Ther..

